# Multivariate time series approaches to extract predictive asthma biomarkers from prospectively patient-collected diary data: a systematic review

**DOI:** 10.1136/bmjopen-2023-079338

**Published:** 2024-08-21

**Authors:** Franz Aaron Apritado Clemeno, Eleanor Quek, Matthew Richardson, Salman Siddiqui

**Affiliations:** 1 Institute for Lung Health, NIHR Leicester Biomedical Research Centre, University of Leicester College of Life Sciences, Leicester, UK; 2 National Heart and Lung Institute, Imperial College London, London, UK

**Keywords:** asthma, telemedicine, patient reported outcome measures, adverse events

## Abstract

**Abstract:**

**Objectives:**

Longitudinal data are common in asthma studies, to assess asthma progression in patients and identify predictors of future outcomes, including asthma exacerbations and asthma control. Different methods can quantify temporal behaviour in prospective patient-collected diary variables to obtain predictive biomarkers of asthma outcomes. The aims of this systematic review were to evaluate methods for extracting biomarkers from longitudinally collected diary data in asthma and investigate associations between them and patient-reported outcomes (PROs) of patients with asthma.

**Design:**

Systematic review and narrative synthesis.

**Data sources:**

MEDLINE, EMBASE, CINAHL and the Cochrane Library were searched for studies published between January 2000 and July 2023.

**Eligibility criteria:**

Included studies generated biomarkers from prospective patient-collected peak expiratory flow, symptom scores, reliever use and nocturnal awakenings, and evaluated their associations with asthma PROs, namely asthma exacerbations, asthma control, asthma-related quality of life and asthma severity.

**Data extraction and synthesis:**

Two independent reviewers used standardised methods to screen and extract data from included studies. Study quality and risk of bias were assessed using the Transparent Reporting of a multivariable prediction model for Individual Prognosis Or Diagnosis (TRIPOD) and the Prediction model Risk Of Bias ASessment Tool (PROBAST), respectively.

**Results:**

24 full-text articles met the inclusion criteria and were included in the review. Generally, higher levels of variability in the diary variables were associated with poorer outcomes, especially increased asthma exacerbation risk, and poor asthma control. There was increasing interest in non-parametric methods to quantify complex behaviour of diary variables (6/24). TRIPOD and PROBAST highlighted a lack of consistent reporting of model performance measures and potential for model bias.

**Conclusion:**

Prospectively patient-collected diary variables aid in generating asthma assessment tools, including surrogate endpoints, for clinical trials and predictive biomarkers of adverse outcomes, warranting remote monitoring. Studies consistently lacked robust reporting of model performance. Future research should use diary variable-derived biomarkers.

**Strengths and limitations of this study**

- This systematic review included all types of studies, including randomised controlled trials, retrospective, open label, non-interventional, and so on, to evaluate the overall utility of prospective patient-collected diary data, beyond interventional studies.
- The heterogeneity of the included studies, in terms of objectives, variables and outcomes meant that a meta-analysis was not within scope.
- This systematic review searching only four databases may have resulted in some relevant studies not being included.

## Introduction

 Asthma is a heterogeneous, chronic, inflammatory disorder of the airways^1^ that currently affects millions of people worldwide.^2^ It has been shown that there is wide variability between patients with asthma in terms of the manifestations of asthma that they may experience,^3^ giving rise to different subtypes of asthma and potentially making it difficult to identify useful generalisable biomarkers to quantify disease activity.

Numerous outcome measures have been used to assess the state of asthma in patients. These include asthma exacerbation occurrence or risk, asthma control, asthma severity and asthma-related and general health-related quality of life.

On top of these, numerous diary variables are often frequently captured longitudinally in studies to assist with asthma monitoring. Symptom scores are frequently collected as a diary variable. Symptom burden and perception can vary widely between patients, and there is a wide range of symptom scoring systems that are used across clinical trials,^4^ which often encompass different items and domains (eg, dyspnoea, wheeze, cough, chest tightness and their frequency/intensity or impact on activities). Peak expiratory flow (PEF), which is the maximal flow rate achieved when performing a forced exhalation from full inspiration, is a common variable to capture airflow obstruction at home using a cheap, portable and easy to use instrument. Personalised asthma action plans frequently use percentage of best PEF alongside self-assessment of symptoms as a prompt for intervention to prevent exacerbations.^5^ Reliever use is another parameter that is frequently diarised. Increased use of short-acting beta agonist has been associated with an increased risk of severe exacerbation.^6^ In clinical trials this is usually reported as number of puffs per day, or number of reliever-free days. Nocturnal awakenings are also often measured in asthma, since it can lead to frequent arousals during sleep and poor sleep quality, with many proposed potential mechanisms^7^ and has been linked to worse asthma control and asthma quality of life. Nocturnal awakening can be monitored in several different ways including fitness trackers, cough recording and sleep position monitors. The use of fractional exhaled nitric oxide (FeNO), a surrogate marker for eosinophilic airway inflammation, as a diary variable was also investigated. There is increasing evidence to suggest that it has utility in predicting corticosteroid responsiveness and adherence, and that using a FeNO-guided medication strategy helps to reduce exacerbations.^8^

Several approaches are available to analyse these time series, to extract biomarkers, to quantify the behaviour of diary variables, which could then be associated with and/or predictive of asthma outcomes. This includes simple measures, like mean and variance, non-parametric methods such as detrended fluctuation analysis (DFA),^9^ which are well-suited for time series data, and machine learning (ML) approaches, which can make use of rich, complex data. Extracting meaningful biomarkers from diary data is useful, since those variables are relatively easy and cheap to measure and record, as opposed to genomic or imaging data. Second, they can also be recorded with a high temporal granularity, thus giving a clear image in the longitudinal behaviour of the diary variables and disease progression.

Previous studies have demonstrated the utility of diary data monitoring to identify trends in disease activity that can be used for intervention in an actionable window to reduce exacerbations, notably the FACET (Formot- erol and Corticosteroids Establishing Therapy) study which showed a gradual and then precipitous decline in peak flow in the days before exacerbation, in conjunction with an increase in symptoms and short acting bronchodilator use.^10^ The efficacy of early intervention was later demonstrated by the FOURFOLD study which showed that temporary quadrupling of inhaled corticosteroid dose when asthma control starts to deteriorate significantly decreased exacerbations compared with standard treatment.^11^

The rising availability of mobile health technologies, otherwise known as 'mHealth', such as digital apps, spirometers, inhalers and remote sensors enables more convenient recording for patients at home of multiple physiological, symptom, environmental and treatment adherence parameters. This has the potential to generate a wealth of data that can be leveraged to generate prediction algorithm tools to facilitate real-time feedback and personalised advice for self-management. Previous systematic reviews on the impact of mHealth interventions for asthma self-management have demonstrated improvements in treatment adherence, quality of life, asthma control and unscheduled emergency department visits compared with standard treatment.^12^

Currently, no systematic review has been undertaken to assess the methodology applied to temporal diary variables, and the associations between the extracted measures and various patient-reported outcomes (PROs) in asthma studies.

The objectives of this systematic review were to: (1) review the methods used to extract biomarkers from longitudinally collected diary data, and (2) report the associations between extracted measures and various asthma PROs.

## Methods

### Data sources and search criteria

Four databases were searched in July 2023 namely Medical Literature Analysis and Retrieval System Online (MEDLINE), The Cochrane Library, Embase and Cumulative Index of Nursing and Allied Healthy Literature Plus (CINAHL). Only studies published from January 2000 and July 2023 were included in the review. The search strategy is provided as online supplemental material.

Further details on methodology can be found in PROSPERO (CRD42021238910).

Keywords and MeSH terms included ‘asthma’, ‘peak expiratory flow’, ‘symptoms’, ‘reliever use’, ‘fractional exhaled nitric oxide’, ‘awakenings’, ‘exacerbations’, ‘control’, ‘severity’, and ‘quality of life’. The full search strategy used is outlined in online supplemental appendix 1.

### Inclusion and exclusion criteria

The review included studies of any type, such as longitudinal, randomised controlled trials, open-label, retrospective analyses, and so on.

The review only evaluated studies that used prospectively patient-collected data in their analyses with (1) a frequency of at least once daily over the course of (2) at least 2 weeks. Studies with a data collection frequency less than mentioned, or that had a shorter duration of follow-up were excluded from the review. This was to ensure that the diary variable data had sufficient temporal resolution and sampling duration to be able to realistically derive predictors of asthma outcomes, without excluding a large number of studies; previous studies that have reviewed fractal analyses of time series highlighted an increased risk of bias in shorter time series (ie, less than 2^6^ points).^13^ Additionally, the data collection frequency mirrors the guideline recommendations for short-term PEF monitoring, for measuring treatment response and establishing baseline values for asthma action plans.^14^

The review only included studies whose participants were adults and/or children of school age (≥5 years old), with clinician-diagnosed asthma. Studies that focused on preschool children were excluded, to avoid confounding asthma with multi-factorial preschool wheeze.

### Review process

Using the predefined inclusion and exclusion criteria, studies were initially selected from their title and abstracts, where a random subset (10%) of these studies were independently reviewed by a second reviewer. Title and abstract screening was performed using Rayyan software.^15^ The full texts of these studies were then obtained, using either interlibrary or British Library requests, were relevant. The full texts were again screened with the same inclusion criteria, where the reasons for exclusions were recorded. The reference lists included were scrutinised to identify further relevant studies.

### Data extraction

Data were extracted from the relevant studies manually, using a prepiloted data extraction form, which included information regarding patient characteristics, diary variables of interest and their corresponding measures. Specifically, the study duration, sample size, the outcome variables and/or endpoints assessed, the diary variables used (from peak flow, night-time awakenings, reliever use and FeNO), the method/s of analysis and markers used in the analysis and the summary of their findings.

### Study quality assessment

Assessment of risk of bias and quality of the included studies were conducted using PROBAST (Prediction model Risk Of Bias ASsessment Tool)^16^ and the Transparent Reporting of a multivariable prediction model for individual prognosis or diagnosis (TRIPOD) checklist,^17^ respectively.

When the study quality was assessed, the percentage adherence of the studies to the different criteria of TRIPOD, as well as overall adherence of the studies to each criterion were recorded, to identify potential sources of bias.

### Patient and public involvement

No patients were involved in the study.

## Results

### Studies identified

**Figure 1:**
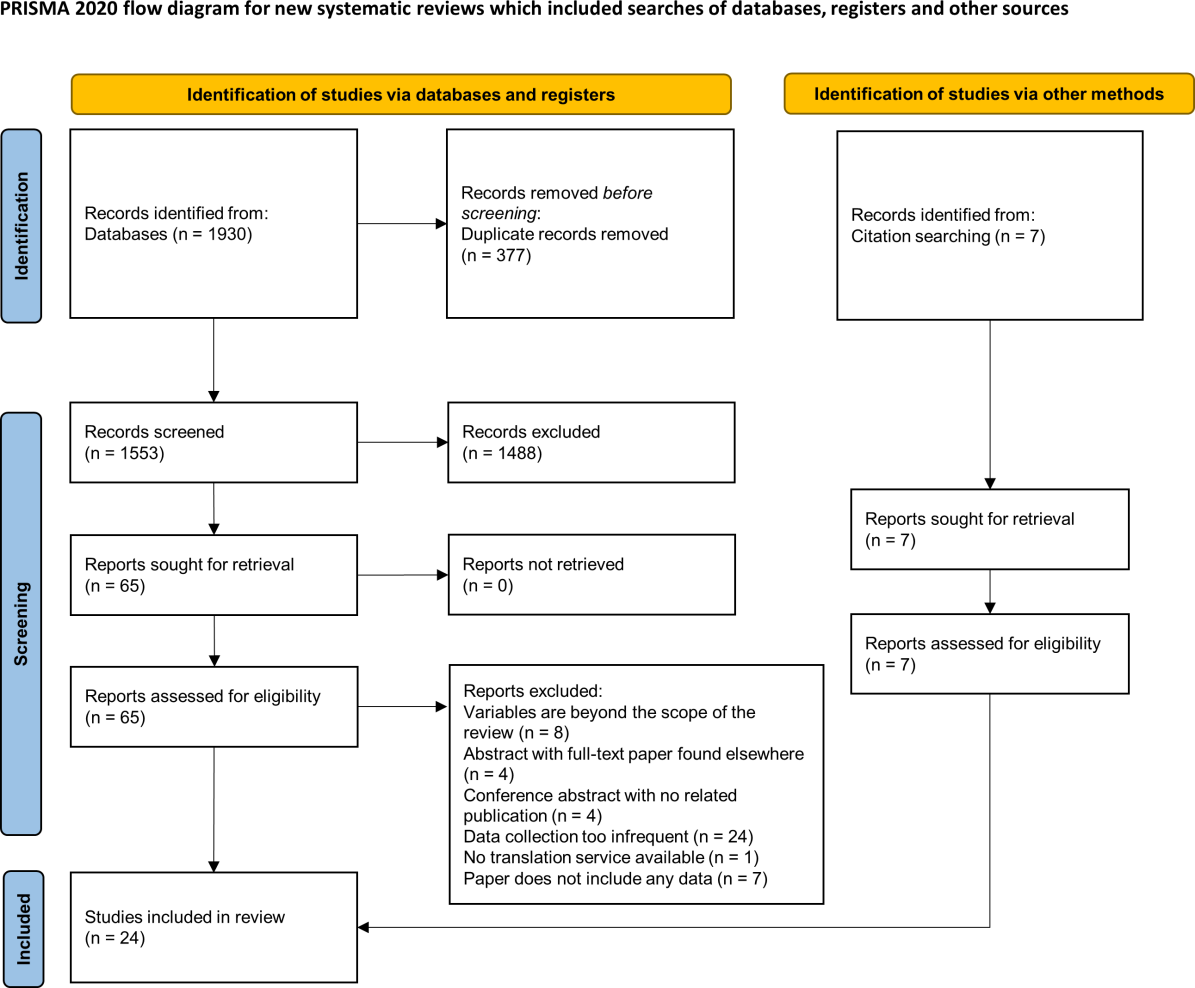
Preferred Reporting Items for Systematic Reviews and Meta-Analyses flow chart illustrating study selection (modified from Page *et al*).^19^

The literature search yielded 1930 results across the four databases, of which 377 were excluded since they were duplicate studies. The remaining titles and abstracts were screened and narrowed down to 65 results for which full-text articles were sought. Using the predefined selection criteria, 48 of these results were excluded. Reasons for exclusion included conference abstract with no related full-text publication (n=4), conference abstract with the full text included elsewhere in the literature search (n=4), studies with data collection being too infrequent (ie, less than at least once-daily over the course of at least 2 weeks) (n=24), publication did not include any data (n=7), studies where the variables were beyond the scope of the review (eg, studies analysed forced expiratory volume in one second (FEV1), immunological markers, airway impedance, etc) (n=8) or a publication was in a language such that translation services were not available (n=1). This left 17 studies for inclusion to the review. Their bibliographies were also searched for relevant papers, from which an additional six studies were identified for inclusion. Additionally, the bibliography of a systematic review that summarised the use of artificial intelligence (AI) in asthma^18^ was searched for potentially relevant studies, and yielded one additional study. Overall, there were 24 studies included in the review. A Preferred Reporting Items for Systematic Reviews and Meta-Analyses (PRISMA) flow diagram^19^ is shown in figure 1. Online supplemental table 1 summarises the 24 included studies.

### Diary variable usage

**Figure 2:**
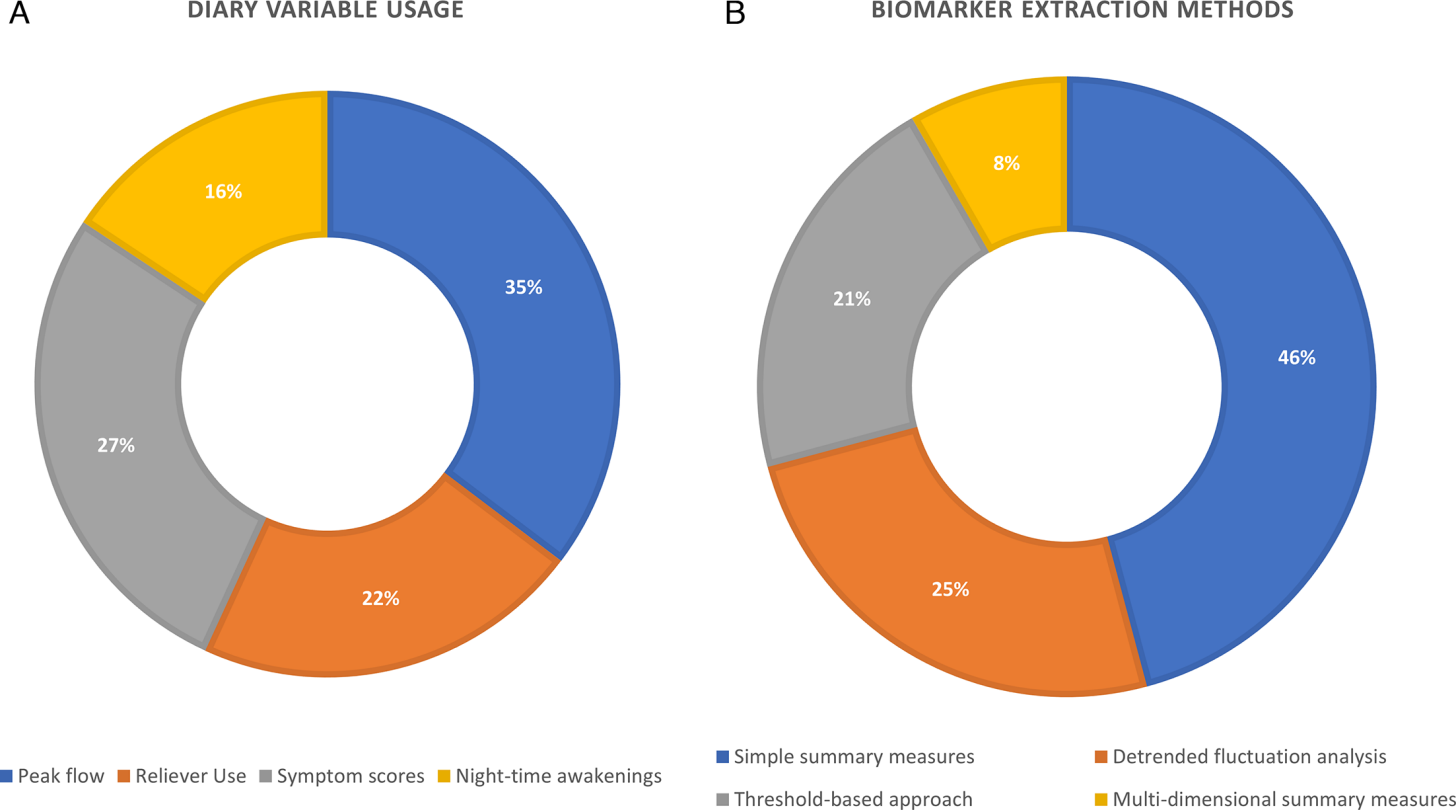
Breakdowns of the included studies by (A) diary variable usage and (B) biomarker extraction methods.

The usage of diary variables in the studies are shown in figure 2A. From the included studies, PEF was the most used diary variable, with 18 studies including it in their analyses. Conversely, night-time awakenings were the least used, with only eight studies using it in their analyses. Symptom scores and short-acting bronchodilator reliever use were used in 14 and 11 of the studies, respectively. Nine of the included studies used only one diary variable in their analyses, and six used all of four. FeNO was included in four of the studies, where it was used as a diary variable in three of them.

### Biomarker extraction methods

The methods used to extract biomarkers from diary variables are summarised in figure 2B. Several studies used simple summary measures to quantify the behaviour of the diary variables throughout the observation periods. These include moving averages,^20 21^ diurnal variability,^22^ seasonal/periodic averages,^23^,^27^ coefficient of variation^26 28 29^ and autocorrelation.^29^ Overall, these studies showed that increased variability in the diary variables is associated with more adverse outcomes, namely exacerbation risk, loss of asthma control and treatment failure to inhaled steroids. Additionally, higher levels of symptom scores, reliever use and night-time awakenings were also associated with increased exacerbation risk or occurrence, and poorer asthma control. Conversely, decreases in PEF were associated with increased exacerbation risk or occurrence. The cross-correlation between daily FeNO and symptom scores were also associated with moderate exacerbation risk, where stronger correlations between the two variables was associated with increased risk.^29^

A non-parametric approach, DFA was used in six of the studies, five of which applied it to time series of PEF recordings, and one to time series of FeNO measurements. DFA quantifies the strength of long-range correlations in the time series through the resulting long-range scaling coefficient, denoted by α. Four of the PEF studies^2830^,^32^ used DFA to extract biomarkers as potential predictors of asthma PROs and the other^33^ solely used DFA to simulate additional PEF time series. These studies show that α is related to asthma PROs, specifically the risk of exacerbations and airway obstruction. Some studies report that a lower α is indicative of increased risk of airway obstruction,^30^ but some found that higher values may be indicative risk of treatment failure to inhaled steroids, when coupled with an increase in the coefficient of variation of PEF.^28^ Lower α values were also found in patients with uncontrolled asthma, but α values did not differ significantly between asthma severity groups.^32^ The DFA coefficient α from PEF during the placebo period was also shown to predict treatment response to salmeterol, but notably, not salbutamol, where higher values of α during the placebo period was associated with improved treatment response. DFA was also applied to time series of FeNO data, and one study found significantly increased α in patients who had experienced an exacerbation.^34^

Several studies used prespecified threshold changes in diary variables over prespecified windows of time to develop markers, and surrogate or early endpoints of asthma PROs. Fuhlbrigge *et al* aimed to develop an intermediate endpoint for asthma exacerbations using diary variables.^35^ The endpoint was defined based on prespecified threshold changes or worsening (slope) greater than some prespecified magnitude, over at least 2 or 5 days, respectively. These thresholds were amalgamated with the American Thoacic Society (ATS)/European Respiratory Society (ERS) definition of asthma exacerbations, defined by oral steroid treatment utilisation,^3^ yielding a composite score. The final endpoint, denoted by CompEx only included PEF, reliever use and symptom scores (CompEx-PRS). CompEx-PRS identified an increased exacerbation event frequency by 2.8-fold, while preserving treatment effect sizes observed on exacerbations.

Kupczyk *et al* also used multiple diary variables and aimed to find a proxy for exacerbations.^36^ A 20% decrease in PEF or a 20% increase in day symptoms on two consecutive days was able to detect severe exacerbations with a sensitivity of 65% and a specificity of 95%, where combining the two improved the overall predictive performance.

Honkoop *et al* aimed to validate optimal action points of PEF and symptoms to aid with early detection of exacerbations.^37^ The optimal combination (PEF and symptoms) action point comprised an increase of more than 2SD of the symptom score from the run-in mean, and a decrease of PEF to <70% of their personal best. This action point detected exacerbations 1.4 days before their occurrence with 80.5% sensitivity and 98.3% specificity.

Wu *et al* used simple thresholds to aggregate daily diary card scores into a symptom score for each 4-month block and evaluated its associations with severe exacerbation occurrence.^27^ Symptom scores were associated with severe exacerbations, where patients with more blocks of persistent symptoms being more likely to experience more exacerbations during the 4-year study.

Spencer *et al* validated a composite measure of asthma control.^38^ The measure was comprised of daytime symptom score, rescue beta2- agonist use, morning PEF, night-time awakening, asthma exacerbations, emergency visits and treatment-related adverse events, and used simple prespecified thresholds to determine asthma control level. The resulting measure showed good discriminative ability of other measures of asthma control, both cross-sectionally and longitudinally.

Van Vliet *et al* compared two methods for assessing asthma control, namely prospective symptom and lung function monitoring versus retrospective recall using the Asthma Control Questionnaire (ACQ).^39^ Prospective assessment of asthma control was measured using daily symptom questionnaires and FEV1 values and using thresholds to classify the level of asthma control on a weekly basis, based on Global Initiative for Asthma (GINA) control criteria.^40^ Conversely, retrospective assessment of asthma control was conducted using the ACQ during the routine clinic visits. There was low concordance between the two methods, but it seems that prospective monitoring provides a more realistic image of patient health, potentially since it minimises recall bias for retrospective recall.

Frey *et al*^30^ and Thamrin *et al*^33^ both used threshold changes of PEF to calculate the conditional probability of an airway obstruction, defined as PEF <80% (moderate) or PEF <60% (severe) of the age-predicted and height-predicted normal values that occur within a certain time period,^40^ given a patient’s current PEF value, denoted by π. As previously mentioned, Frey *et al* found that airway obstruction risk was associated with increased variability and loss of deterministic behaviour of PEF. Thamrin *et al* found that π was associated with actual occurrences of airway obstructions. Additionally, π was shown to be associated with future exacerbation risk, where an increase in this probability was associated with an increase in the OR of having a future exacerbation.

Greenberg *et al* used a threshold-based approach to develop a composite score, named ADAS-6, comprised of rescue beta-agonist use (daily use and diurnal variability), PEF diurnal variability and night-time awakenings, as well as FEV1 % predicted and AQLQ (symptom domain score), to determine the level of disease activity in patients.^41^ The authors defined disease activity based on high and/or low cut-offs for the following variables: daytime symptom score, night-time awakenings, average rescue beta-agonist use, AQLQ score (activity domain), FEV1 % predicted, and asthma attacks. ADAS-6 was discriminative of disease activity and demonstrated content and convergent validity. The study found that each of the six included variables contributed to the regression models in a relatively, balanced manner, looking at their standardised coefficients.

Of the studies in the review, four analysed data, including the diary variables of interest using ML and AI. These studies aimed to build predictive models. Several algorithms were used, namely ensemble learning, Naïve Bayes, support vector machines (SVMs), adaptive Bayesian networks, XGBoost, one class SVM, logistic regression, decision trees and perceptrons. ML models demonstrated good predictive performance in the studies.

Khasha *et al* used an ensemble model, which combined numerous disease-related variables and and medical knowledge to detect asthma control level, which was determined using a rule-based classifier derived from the physicians’ knowledge.^42^ The resulting classifier had a good performance, with an accuracy of over 91%. Interestingly, among the large number of variables used in the algorithm, morning and evening PEF, along with ACT score, as a measure of daily symptoms, were the most important features.

Finkelstein and Jeong used diary data collected through telemonitoring and evaluated three ML methods to build a predictive model for early prediction of asthma exacerbations.^43^ The authors used a naïve Bayesian classifier, adaptive Bayesian network and SVM, of which the adaptive Bayesian network performed best, resulting in a perfect classification in terms of sensitivity, specificity and accuracy when a 7-day window was used to predict an exacerbation on the eighth day.

Zhang *et al* was also interested in predicting exacerbation occurrence using daily diary data, but whether an exacerbation occurred on the same day or up to 3 days in the future.^44^ The authors evaluated the performance of several ML methods, namely, logistic regression, decision tree, naïve Bayes classifier and perceptron algorithms. The best performing model was logistic regression applied to data processed using principal component analysis, and achieved ROC=0.85, sensitivity=90%, specificity=83% for detecting severe asthma exacerbations.

De Hond *et al*^45^ developed and compared predictive models for the early detection of severe asthma exacerbations, using a 2-day prediction horizon. The authors compared the performances of two ML models (XGBoost and one class SVM), a logistic regression model and a simple asthma action plan. The logistic regression model (AUC=0.88) outperformed the XGBoost model (AUC=0.81), as well as the one class SVM model. Notably, both the XGBoost and logistic regression models reached higher discriminative performance compared with the simple clinical rule.

With the extracted biomarkers, regression was the most utilised class of methods for assessing their associations with the PROs. Of the included studies, 15 used a regression method in their analyses. These include many different classes of models, including linear, multinomial, random effects, Cox, etc. A few studies used more complex regression models, such as repeated time-to-event analysis^21^ and generalised estimating equations.^27^

### Risk of bias and quality assessment

**Figure 3:**
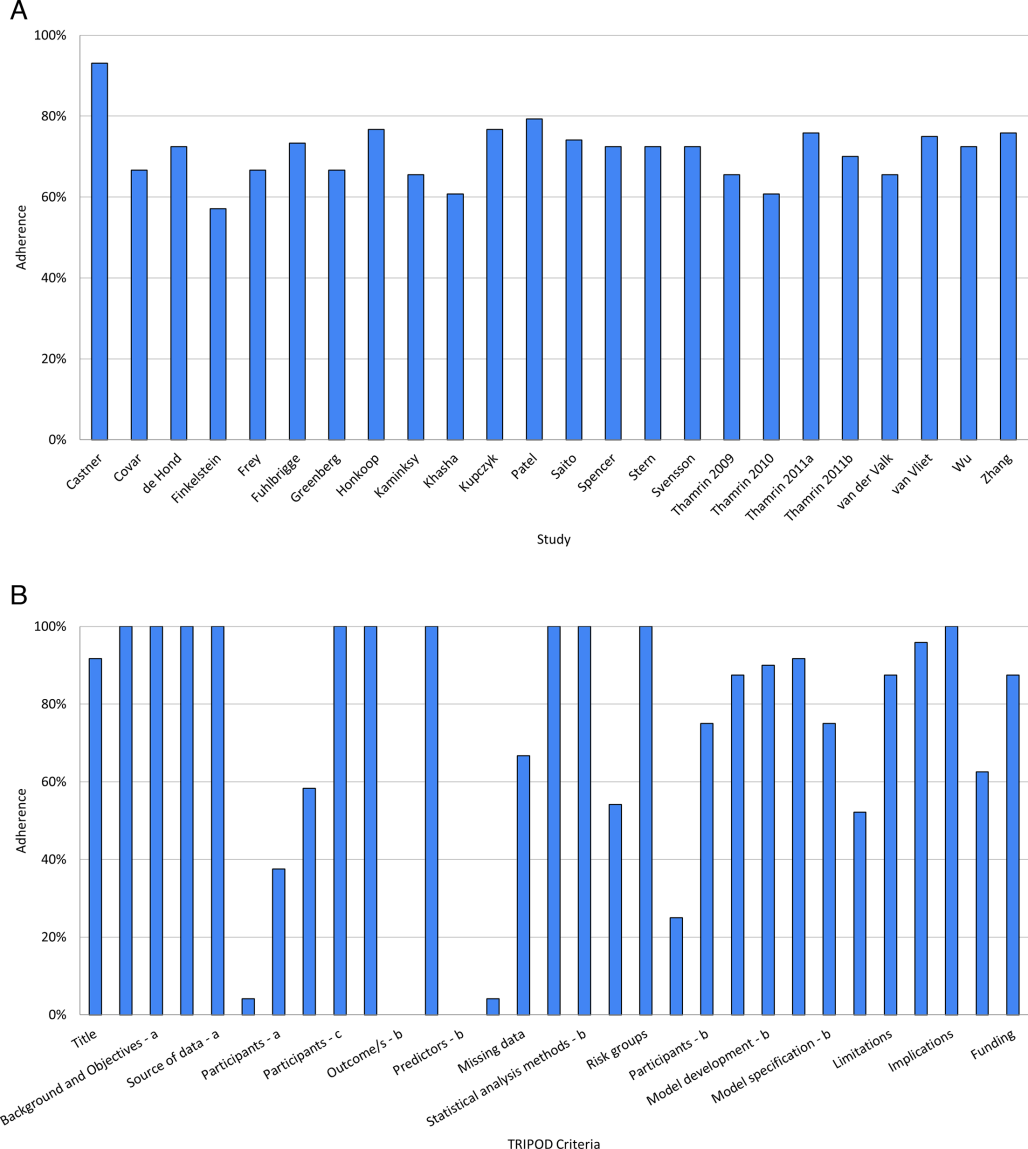
Percentage compliance to TRIPOD checklist of the included studies, broken down by (**A**) study and (B) separate criteria. TRIPOD, Transparent Reporting of a multivariable prediction model for Individual Prognosis Or Diagnosis.

None of the studies showed full compliance to the TRIPOD reporting criteria. The percentage adherence of reporting to the TRIPOD criteria is shown in figure 3, broken down by study (figure 3A) and by criterion (figure 3B). Lack of adherence was prominent in the reporting of key elements of the study setting, participant flow and model performance measures, where of the 24 studies, only 9 (38%), 6 (25%) and 12 (52%) studies reported these, respectively. Of the separate criteria, only one of the studies reported the key study dates, specifically the start and end of accrual. Some of these can be attributed to the fact that 16 of the studies were retrospective, and so simply referred to the original publications for that information.

**Figure 4:**
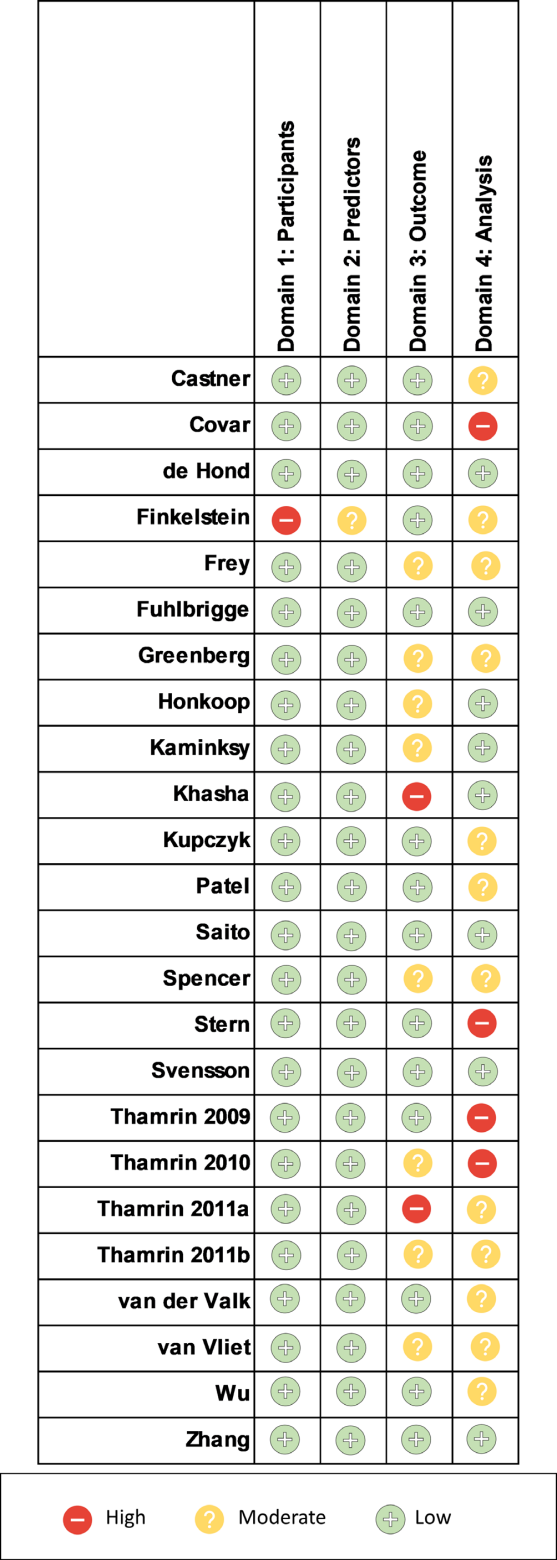
Risk of bias assessment using the Prediction model Risk Of Bias ASessment Tool signalling questions.

Assessment of risk of bias using PROBAST can be found in figure 4. One prominent source of bias among the included studies were in Domain 4: Analysis. Many studies did not appropriately report model performance measures, specifically regarding model overfitting, underfitting and optimism, where only seven studies reported the appropriate measures. This finding is confirmatory of those observed when looking at study adherence to TRIPOD. None of the included studies were deemed to have a high risk of bias overall.

Another source of potential bias that is not addressed in the PROBAST is recording bias, which comes from studies not using electronic or automated method to record diary variables. Ten (42%) of the 24 studies recorded patient data on paper diaries.

## Discussion

This systematic review provided an overview of methodologies applied to prospective patient-collected diary variables in adult asthma, namely, PEF, FeNO, reliever use, night-time awakenings and symptoms to extract biomarkers of PROs, namely asthma exacerbations, asthma control and asthma severity, of which 24 studies were identified. The heterogeneity of the studies, in terms of outcome variables and extracted measures meant that conducting a meta-analysis was not possible.

Of the diary variables of interest, PEF was the most used, potentially because it is both an index of airflow obstruction and the only continuous variable of those included and can hence demonstrate more complex behaviour. Conversely, night-time awakenings were the least used, possibly due to them usually being included as a binary variable. Reliever use and symptom scores were often included as discrete scores, which can display some level of complex temporal behaviour, but not to the extent of PEF. Despite the differences in the type of variables, they demonstrated balanced contributions to asthma disease activity.^41^ Interestingly, only a few studies recorded FeNO as a diary variable. This is despite FeNO being a biomarker of airway inflammation and exhibiting long-range temporal correlations. Additionally, its joint behaviour with symptom scores, quantified by cross-correlation, was significantly associated with asthma exacerbations occurrence.^34^ Further research should investigate complex temporal behaviour of FeNO as potential predictors of asthma PROs.

Few studies used ML and AI models to build predictive models with diary variables, even though prospective patient-collected diary variables can provide numerous data points per patient. The studies that opted to use these methods^42^,^44^ built predictive models which showed decent performance. A systematic review of AI in asthma found a growing interest in the use of such methods, but there is currently a lack of research in the context of asthma treatment, especially to biologics.^18^ One study^45^ however demonstrated that ML models do not always outperform classic statistical models, such as logistic regression, and that researchers should consider whether the complexity of the data warrants the use of complex ML methods. Future studies can use ML models to use the complex temporal behaviour of the data to predict PROs, alongside other data streams simultaneously, such as demographic, genomic data.

The systematic review has demonstrated that biomarkers that quantify the temporal behaviour of diary variables are associated with asthma PROs, namely asthma exacerbations, asthma control and treatment response or failure. Generally, higher levels of variability in the diary variables were associated with increased risk of asthma exacerbations and uncontrolled asthma. Threshold-based approaches were the most common among the included studies for extracting biomarkers from the diary variables. These methods were useful in identifying and quantifying simple temporal changes in the diary variables over a short prespecified period. Threshold approaches resulted in markers that were able to detect the occurrence of asthma exacerbations early. Temporal variability was also quantified using a range of simple summary measures, as well as novel non-parametric methods, such as DFA. DFA first introduced by Peng *et al* and was initially applied to time series of DNA nucleotides^9^ and later to time series of heart rate.^46^ DFA quantifies fractal scaling properties of time series, as well as detecting and quantifying the long-range correlations of non-stationary time series, into one quantitative parameter α. DFA has been applied to numerous physiological time series, such as to detect irregularities in the heart rate of patients with severe congestive heart failure,^47^ as well as to differentiate between healthy and patients with diabetes, the fluctuations in their plasma glucose.^48^ DFA has also been used to identify long-term self-similarity in the daily PEF of patients with COPD, and that α was significantly associated with exacerbation frequency.^49^ This systematic review identified an increasing use of DFA, which quantifies more complex long-range scaling temporal behaviour of the diary variables, compared with simple summary measures and threshold-based approaches. DFA has shown promise in generating biomarkers, which are associated with PROs, including asthma exacerbations and treatment response. Despite being mainly applied to time series of PEF data, application to FeNO data has shown promise and should be further explored in future studies.

Future studies can also employ alternative methods to quantify temporal behaviour. One example is entropy,^50^ which is a method that quantifies the randomness and irregularity of a time series or physiological signal. It has been applied to time series of airway resistance in patients with asthma and found it to be associated with frequent exacerbations.^51^ A systematic review of the applications of entropy in asthma found that the entropy values of signals such as respiratory sound and airway resistance, and respiratory interbreath interval, are strong potential novel indices of asthma progression and asthma severity, respectively^52^; however, no published studies to date have applied entropy to time series of diary variables and evaluated its association with asthma PROs.

For identifying and quantifying associations between the extracted markers and PROs, regression methods were the most common. Regression models have model coefficients and results which are often easy to interpret for researchers and clinicians, and many classes of regression models are available to handle different data types.

The review demonstrates that inclusion of more than one diary variable in analyses showed utility, as different measures capture different aspects of asthma. The temporal relationship between FeNO and symptoms, quantified using cross-correlation was shown to be a potential predictor of exacerbations.^34^ Greenberg *et al* used multiple diary variables to develop a score that measured disease activity and was related to asthma attack occurrence.^41^ Interestingly, the diary variables contributed equally to the score and removing diurnal variability worsened its performance, especially in small sample sizes. Future studies should consider temporal relationships between diary variables as potential predictors of asthma PROs.

The included studies demonstrated partial adherence to TRIPOD criteria, specifically with reporting model performance measures. A systematic review of prediction models for future asthma exacerbations similarly report a lack of robust validation analyses to demonstrate generalisability of results.^53^ Future studies should improve reporting adherence to TRIPOD, to enable qualification of markers as asthma endpoints.

Based on the PROBAST, none of the studies had an overall high risk of bias. One significant source of bias was a lack of sufficient reporting in terms of the model performances in concordance with the TRIPOD analysis. While this does not affect the models’ performances, it helps readers determine the generalisability of the developed models for readers. Another source of potential bias was from manual collection of diary variables. This introduces potential measurement and recall bias, especially if patients were expected to collect and record this data themselves, rather than by a physician.

Another potential source of bias is the consistency and consideration of the data collection times.^54^ Asthma demonstrates significant diurnal rhythmicity, where symptoms generally worsen overnight or early in the morning.^55^ Conversely, night-time PEF and FEV1 are reduced compared with daytime values.^56^ Additionally, studies have shown that there is within-day variability in airway inflammation, measured using FeNO, even in stable asthmatics.^57^ These suggest that timing of the diary variable collection is important, and that consistent timings in the collection are vital in minimising the effect of within-day variability due to circadian rhythms, which would potentially mask fluctuations caused by disease.

Limitations of this systematic review include the exclusion of conference abstracts and the search for studies being limited to four databases.

**Figure 5:**
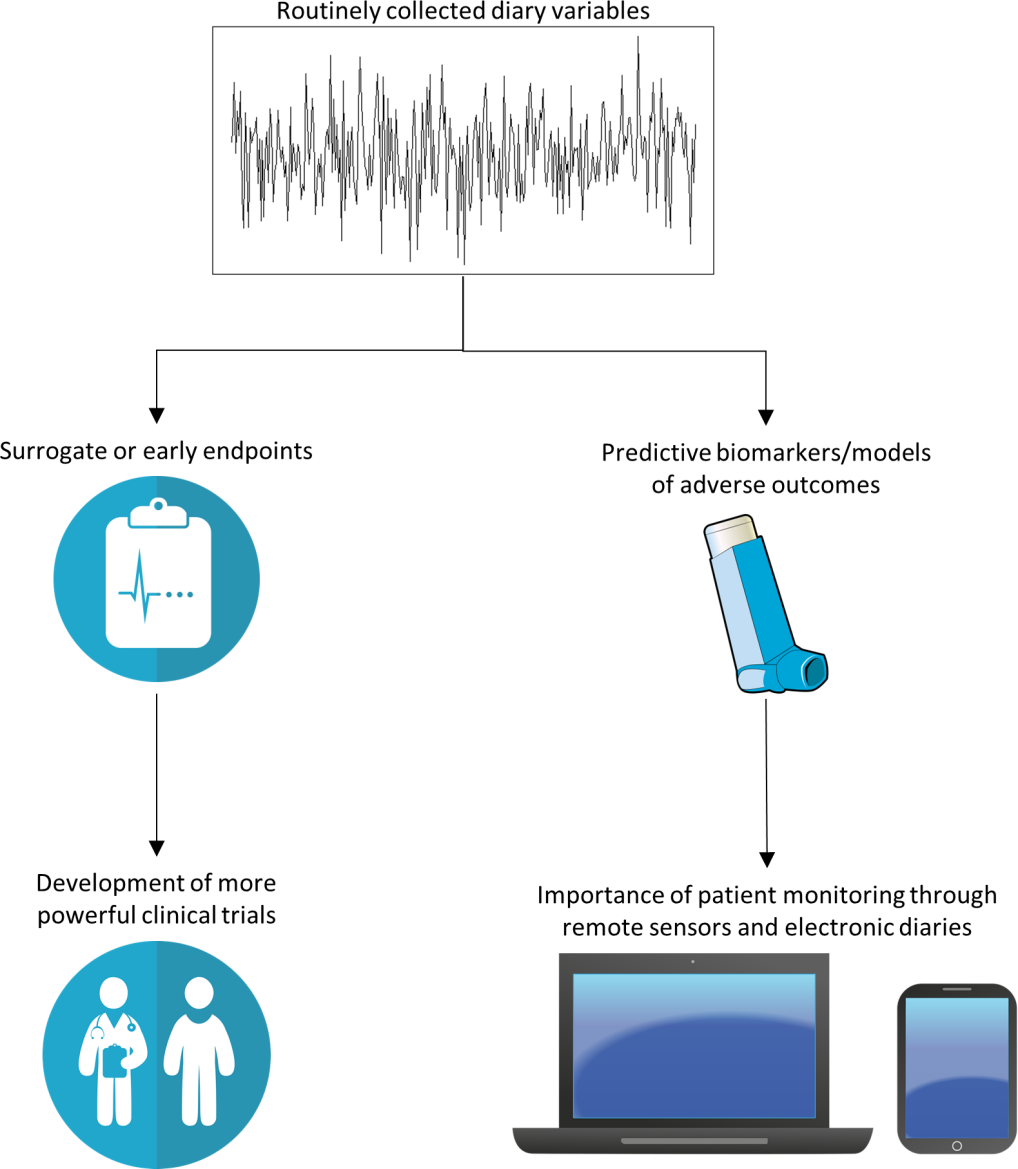
Clinical utility of longitudinally collected diary variables.

The routine use of mobile technology in everyday life means that multidimensional data can be more readily aggregated by patients actively (eg, apps and electronic diaries to record symptom burden, lung function), and passively through remote sensors (eg, digital inhalers, wearable monitors, environmental air quality/pollen count sensors). Our review has shown the ways in which hidden patterns in these vast datasets can be elucidated to predict salient asthma outcomes. The included studies show that prospectively patient-collected diary variables demonstrate clinical utility in two domains: (1) the generation of asthma assessment tools, such as surrogate markers or early endpoints, which can aid researchers and clinicians to design shorter and more powerful clinical trials; (2) the discovery and/or generation of biomarkers or models, which are predictive of adverse outcomes in asthma. In particular, the latter domain may confer the ability to better characterise asthma phenotypes and be used to predict disease trajectories on an individual basis, which could be used to deliver personalised treatment plans (eg, in asthma attack prediction and intervention prompts) and empower patients as active partners in the management of their disease. This is summarised in figure 5.

In summary, this review highlights the importance of quantifying the longitudinal, often complex behaviour of daily-recorded diary variables and their utility in developing biomarkers that are predictive of asthma outcomes, namely asthma exacerbations, asthma control, asthma severity and asthma-related quality of life. Consequently, future research should expand non-parametric methods that can be used to quantify this behaviour, alongside standardising both the capture of diary data, in view of the growing number of digital devices and m-health technology used to acquire diary variables in asthma. Future studies should adhere to robust multivariable model reporting standards, specifically in terms of model performance and validation, to allow for qualification of the biomarkers in the context of interventional studies.

## supplementary material

10.1136/bmjopen-2023-079338 online supplemental file 1

10.1136/bmjopen-2023-079338 online supplemental file 2

## Data Availability

All data relevant to the study are included in the article or uploaded as supplementary information.
