# Supplementary material for: Multivariate time series approaches to extract predictive asthma biomarkers from prospectively patient-collected diary data: a systematic review
Source: BMJ Open. 2024 Aug 21;14(8):e079338. doi: 10.1136/bmjopen-2023-079338 (PMC11340722; doi:10.1136/bmjopen-2023-079338)
Supplement: online supplemental file 1 [file bmjopen-14-8-s001.docx]

| Study | Modelled outcome variable/s | Duration | Sample Size | Diary variables used | Analysis method/s | Summary of findings |
| --- | --- | --- | --- | --- | --- | --- |
| Castner et al [20] | Asthma control | 6-8 weeks | 43 | Symptoms, awakenings | 3-day moving averages were calculated from diary data  Random effects models were used to identify significant predictors | 3-day moving averages of symptom scores were significantly predictive of asthma-specific awakenings and FEV1.  Wake counts were significantly associated with FEV1. |
| Covar et al [23] | Asthma exacerbations | 48 weeks | 285 | PEF, symptoms, reliever use | PEF values were summarised as seasonal averages in three-month blocks.  Regression modelling was used to investigate associations with occurrence of exacerbations.  Logistic regression models were used in the analysis. Univariate models were first built with each of the measures to narrow down the statistically significant covariates (p < 0.05) to include in the final model. | PEF expressed as the average drop in PEF over the entire season was significantly associated with the occurrence of exacerbations.  Diary variables evidently changed 12 to 3 days before an exacerbation, with a more apparent change within the last 2 days. Outcome measures returned to baseline within a maximum period of 10 days. |
| de Hond et al [46] | Asthma exacerbations | Development set: median 610 days  Validation set median 417 days | Development set: 165  Validation set: 101 | PEF, reliever use, awakenings | Twice-daily measured PEF and reliever use, and nocturnal awakenings used as predictors. Various summary statistics (average, standard deviation, maximum and minimum) and time series transformations (first difference and lag) were also included in the models.  The performance of logistic regression was compared with one class SVM and XGBoost models to predict severe asthma exacerbation in a 2-day window. Their performance was compared to a proposed clinical rule (start oral corticosteroids treatment if PEF <60% personal best). | XGBoost achieved an AUC of 0.81, while logistic regression had an AUC of 0.88.  One class SVM had a lower sensitivity (0.34) than both XGBoost and logistic regression (0.6 and 0.73, respectively).  Both XGBoost and logistic regression achieved higher sensitivity than the clinical rule. |
| Finkelstein and Jeong [44] | Asthma exacerbations | Not stated | 26 | PEF, symptom score, reliever use, awakenings | Three classifiers were used: naïve Bayes, adaptive Bayesian network and support vector machines, using a 7-day window to predict exacerbation occurrence on day 8. | Using a 7-day window, the adaptive Bayesian network resulted in a perfect classification (sensitivity and specificity)  Shortening the time window resulted in worsened performance of the ML algorithms. |
| Frey et al [30] | Asthma exacerbations | 18 months | 80 | PEF | DFA was applied to the PEF time series to quantify its temporal behaviour, resulting in α. The coefficient of variation (CV) was also calculated.  The conditional probability of experiencing a significant deterioration in airway obstruction was calculated. Calculation of the conditional probabilities was based on pre-specified threshold changes of PEF.  A nonlinear stochastic model of the fluctuations was introduced to assess the separate effects of the distribution and correlation of the PEF time series on the risk. | Lower α values generally reflect more severe airflow obstructions.  The risk of airway obstruction increases with decreasing α coupled with increased CV.  LABA shown to increase α and decrease risk while SABA does opposite |
| Fuhlbrigge et al [35] | Treatment response | 6/12 months | 17,415 | PEF, reliever use, symptom scores, awakenings | Threshold-based approach to develop event-based surrogate endpoint for severe exacerbations.  Various combinations of the four diary variables were evaluated to identify the optimal combination. Different combinations of the threshold and slope levels were also evaluated to select the most robust algorithm for CompEx.  Optimal CompEx algorithm was then selected so that the hazard ratio (HR) between treatment and control arms in trial was near equivalent to the HR of severe exacerbations | Composite endpoint from PEF, symptom score and reliever use was found to be optimal  Found that CompEx increased significant events by 2.8 times compared to using severe exacerbation as endpoint, while preserving treatment effect, CompEx found to increase power by 67% and reduce sample size needed by more than 50%. Also reduced clinical trial length needed to 3 months |
| Greenberg et al [42] | Asthma exacerbations | 17 weeks | 1,114 | PEF, symptom scores, reliever use, awakenings | Disease activity was based on high and low cutoffs of daytime symptom score, awakenings, average rescue medication use, AQLQ-Activity domain, FEV1 and asthma attacks.  Stepwise, forward multiple regression analysis was used to determine which of the parameters to include in the weighted measure (ADAS-6). | Total β-agonist use/day, PEF and β- agonist use diurnal variability, and night-time awakenings contributed to the disease activity score, in a relatively balanced manner.  ADAS-6 discriminated between different levels of disease activity, as well as showing predictive validity for the risk of future asthma attacks. |
| Honkoop et al [37] | Asthma exacerbations | 1 year | 294 | PEF, symptom scores | Different action points (AP) were formed based on pre-specified thresholds for diary variables. Both univariate and multivariate action points were evaluated. Their performances were quantified using early detection days, sensitivity, specificity, Accuracy, AUC and NNT. | The optimal AP for early detection of asthma exacerbations was an increase in the composite symptoms score by greater than two standard deviations (from run-in) and a fall in PEF to <70% of their personal best occurring within a 1-week window. |
| Kaminsky et al [28] | Treatment response | 4-6 weeks run-in period  16-weeks treatment period | (1) 167  (2) 161  (3) 165 | PEF | The DFA coefficient α and coefficient of variation (CV) were calculated from PEF data during the run-in phase and the treatment phase. | An increase in α coupled with an increased CV is associated with treatment failure.  The pattern of alpha preceding treatment failure varied across the participants. |
| Khasha et al [43] | Asthma control | 9 months | 96 | PEF | Artificial intelligence (AI) model using ensemble learning, incorporating seven multiple base learners along with medical knowledge. Medical knowledge was incorporated through a rule-based classifier to classify the patient’s asthma control level. | Morning and evening PEF were in the top three most important variables for asthma control level detection (alongside ACT score).  The proposed ensemble model had an accuracy of 0.943 for well-controlled zone, 0.894 for not well-controlled zone and 0.913 for very poorly controlled zone. |
| Kupczyk et al [24] | Asthma exacerbations | 1 year | Severe asthma 93  Mild to moderate asthma 76 | PEF, symptom score, reliever use | Looked at percentage changes from baseline (personal best during optimisation phase of study) in diary variables at different periods pre-, during, and post-exacerbations.  ROC curves for different percentage changes in PEF and FEV1 were performed to determine the sensitivity and specificity of different variable cut off points to detect a severe exacerbation | Regular monitoring of diary variables is able to detect severe exacerbations.  A 20% decrease in PEF had a sensitivity of 45% and specificity of 85% for detection of severe exacerbations (SEs)  20% increase in day time symptoms had a sensitivity of 46% and specificity of 84.9% for detection of severe exacerbations (SEs)  An algorithm combining a 20% decrease in PEF or a 20% increase in day symptoms on 2 consecutive days was able to detect SEs with 65% sensitivity and 95% specificity. |
| Patel et al [25] | Asthma exacerbations, asthma control | 24 weeks | 147 in exacerbation analysis  142 in asthma control analysis | Reliever (salbutamol) use | Baseline reliever use metrics were used in the analysis.  Logistic regression was used to estimate the odds ratio for the association between the various metrics of salbutamol use and risk of severe asthma exacerbations, poor asthma control, and extreme salbutamol overuse. | Higher mean daily reliever, higher days of reliever, and higher maximal 24-hour use were associated with future severe exacerbations.  Higher mean daily use was associated with poor asthma control |
| Saito et al [22] | Asthma control, asthma severity | 2 weeks | 65 | PEF, FeNO | Diurnal variation, as well as other measures of daily and weekly variability was calculated for FeNO and PEF time series.  ROC curves were used to determine the detection of uncontrolled asthma.  Multivariate logistic regression analysis was used to identify predictors of uncontrolled asthma. | Diurnal variation of FeNO was able to discriminate between levels of asthma control, but diurnal variability of PEF was not able to.  Neither PEF or FeNO diurnal variability were able to discriminate between levels of asthma severity. |
| Spencer et al [38] | Asthma control | 1 year | 3,416 | PEF, reliever use, symptom scores, awakenings | Pre-specified thresholds of daytime symptom score, rescue beta2-agonist use, morning PEF, night-time awakening, asthma exacerbations, emergency visits, and treatment-related adverse events were used to determine the level of control each week (totally controlled (TC) or well-controlled (WC)).  Its relationship with the reference criteria (FEV1 and AQLQ) was tested using logistic regression models. | TC and WC asthma showed good discriminative properties when compared with % pred FEV1 and AQLQ at week 12 and change in % pred FEV1 from baseline to Week 52.  The composite asthma control measures have better discriminative properties compared to the individual asthma control status components alone. |
| Stern et al [34] | Asthma control, asthma exacerbations | 30 weeks | 41 | Symptoms, FeNO | DFA was applied to FeNO time series, resulting in the long-range scaling coefficient α. Cross-correlation was calculated to quantify the linear correlation between FeNO and symptom scores.  Associations between the measures and outcomes of interest were evaluated using linear regression analysis. | Daily fluctuations in FENO values exhibited fractal-type long-range correlations. α values were significantly associated with baseline ICS use but were not associated with asthma control averaged over the whole period or in the last 12 weeks of the study.  Both α values and cross-correlation were able to distinguish between patients who experienced an exacerbation and those who did not. The cross-correlation between FeNO values and symptom scores was significantly higher in those subjects who had exacerbations. |
| Svensson et al [21] | Asthma exacerbations | 52 weeks | 502 | PEF, reliever use, symptom scores, awakenings | Diary variables were summarized as 4-day averages.  Stepwise covariate model selection was used to determine relevant predictors.  An overall repeated time-to-event (RTTE) analysis was used to model repeated event data, which incorporates baseline and time-varying covariates, as well as treatment exposure and baseline hazard. | Diary variables showed trends 10-20 days prior to exacerbation events. PEF decreased and symptoms, reliever use, and awakenings increased.    Symptom score and rescue medication were significant in the model as time-varying covariates.    PEF was significant in the forward selection but not in the backward selection. |
| Thamrin et al 2009 [31] | Treatment response | 6 months for each treatment period | 66 | PEF, symptom score | DFA was applied to PEF time series, using only 300 data points for each treatment period, which corresponds to the first 150 days of each treatment period. The resulting value is denoted α.  Regression models used to examine associations between predictors of interest and the clinical outcomes. | α calculated from the placebo period was significantly associated with treatment response to salmeterol, where higher values suggested a decrease in symptom days. |
| Thamrin et al 2010 [26] | Asthma control | 2 weeks pre-withdrawal period  6 weeks or until loss of control post-withdrawal, whichever came first | 83 | PEF | Variability measures were calculated both pre- and post-withdrawal, including the coefficient of variation of PEF (CV).  Cox regression was used to explore associations between time to loss of asthma control (LOC) and the variability measures. ROC curves were used to assess the utility of each of the measures for predicting LOC. | An increase in CV in any of the three periods were significant predictors of LOC in the separate models. Autocorrelation was not a significant predictor of LOC, even when the trend was removed.  The larger the increase in CV within 2 weeks of ICS withdrawal, the sooner the patient will experience LOC. |
| Thamrin et al 2011a [33] | Asthma exacerbations | Study A: 52 weeks  Study B: 72 weeks | Study A: 77  Study B: 58 | PEF | The conditional probability is calculated from simulated PEF time series, using DFA to quantify the correlation properties.  Calculation of the conditional probabilities was based on pre-specified threshold changes of PEF.  Logistic regression was used to examine associations between conditional probabilities and PEF events and asthma exacerbations. | The conditional probability was related to actual decreases in PEF. A 10% increase in the probability was associated with the risk of having a future exacerbation. |
| Thamrin et al 2011b [32] | Asthma control, asthma exacerbations, asthma severity | 6 months | Study 1: 132  Study 2: 159 | PEF | DFA was performed on twice-daily PEF to derive α.  Associations between α and % predicted PEF and outcome variables examined with binary or multinomial logistic regression. | α values were able to discriminate between levels of asthma control, where lower values were found in patients with uncontrolled asthma.  α values did not differ significantly between severity groups.  Patients with exacerbations had significantly higher α values than those who did not. |
| Van der Valk et al [29] | Asthma exacerbations | 30 weeks | 27 | Reliever use, FeNO | Daily FeNO and symptom scores were taken from 3-week blocks. Fluctuation and correlation metrics used were coefficient of variation, the slope of daily FeNO, cross-correlation and autocorrelation.  Parameters were evaluated as predictors for exacerbations using logistic regression. | The following FeNO parameters were associated with moderate exacerbation risk:  - CV (14-10 days before)  - Slope (14-4 days before)  - Cross-correlation (14-10 days before)  - Autocorrelation (21-10 days before)  There was marked variability in FeNO but no clear rise preceding the onset of severe exacerbations.  There was no clear trend of symptoms relative to severe exacerbations. |
| Van Vliet et al [39] | Asthma control | 1 year | 78 | Symptoms | Asthma control by home monitoring was determined based on data 1 week before the clinical visit and was calculated based on GINA criteria for asthma control (threshold-based approach).  Agreement between the two methods was analyzed by the linear Cohen kappa coefficient. | There was low agreement between the 2 instruments to distinguish the 3 levels of asthma control. |
| Wu et al [27] | Asthma exacerbations | 4 years | 1,019 | Symptoms | Symptom scores were aggregated in four-month blocks. Pre-specified thresholds were used to classify patients as to whether they experienced persistent symptoms.  Generalized estimating equation (GEE) models were used to study predictors of persistent symptoms, as well as severe exacerbations. | Symptom category is associated with severe exacerbations even when adjusting for demographic, pulmonary and biologic measures.  Predictors of both persistent symptoms and severe exacerbations include ICS treatment (budenoside), FEV1/FVC ratio, and PC20. |
| Zhang et al [45] | Asthma exacerbations | Mean 362 days | 2,010 | PEF, reliever use, symptom scores, awakenings | Four machine learning models were trained and tested, namely logistic regression, naïve Bayes, decision trees and perceptrons. | The best model used logistic regression with input variables derived from principal components analysis.  The model had an area under the receiver operating characteristic curve of 0.85, with a sensitivity of 90% and specificity of 83% for detecting severe asthma exacerbations up to three days before its occurrence. |

Table 1: Summary table of the included studies. Abbreviations used: PEF = peak expiratory flow; FeNO = fractional exhaled nitric oxide; DFA = detrended fluctuation analysis; AUC = area under curve; CV = coefficient of variation; AI = artificial intelligence; ROC = receiver operating characteristic; FEV1 = forced expiratory volume in 1 second; AQLQ = Asthma Quality of Life Questionnaire; ACT = Asthma Control Test; ICS = inhaled corticosteroid.
