# Supplementary material for: Multivariate time series approaches to extract predictive asthma biomarkers from prospectively patient-collected diary data: a systematic review
Source: BMJ Open. 2024 Aug 21;14(8):e079338. doi: 10.1136/bmjopen-2023-079338 (PMC11340722; doi:10.1136/bmjopen-2023-079338)
Supplement: online supplemental file 2 [file bmjopen-14-8-s002.pdf]

Appendix 1

The search strategy that was used for all four databases (EMBASE, MEDLINE, CINAHL, The Cochrane Library) is outlined below:

1. exp Asthma/
2. asthma*.mp.
3. 1 or 2
4. "diary variable".mp.
5. "diary data".mp.
6. "lung function".mp.
7. Peak Expiratory Flow Rate/
8. "peak expiratory flow".mp.
9. "reliever use".mp.
10. "rescue medication".mp.
11. "inhaler use".mp.
12. awakening*.mp.
13. symptom score*.mp.
14. "airway inflammation".mp.
15. Biomarkers/
16. fractional exhaled nitric oxide.mp.
17. Symptom Flare Up/
18. symptom*.mp.
19. 4 or 5
20. 7 or 8
21. 9 or 10 or 11

22. 13 or 17 or 18
23. 14 or 15 or 16
24. 6 or 12 or 19 or 20 or 21 or 22 or 23
25. Patient Reported Outcome Measures/
26. patient reported outcome*.mp.
27. exacerbation*.mp.
28. asthma attack*.mp.
29. Airway Obstruction/
30. "airway obstruction".mp.
31. "airway deterioration".mp.
32. "asthma episode".mp.
33. "Quality of Life"/
34. "quality of life".mp.
35. "Asthma control".mp.
36. "asthma severity".mp.
37. 25 or 26
38. 27 or 28 or 29 or 30 or 31 or 32
39. 33 or 34
40. 35 or 36 or 37 or 38 or 39
41. 3 and 24 and 40
42. limit 41 to yr="2000 -Current"
43. limit 42 to english language
