# Supplementary material for: Multivariate time series approaches to extract predictive asthma biomarkers from prospectively patient-collected diary data: a systematic review
Source: BMJ Open. 2024 Aug 21;14(8):e079338. doi: 10.1136/bmjopen-2023-079338 (PMC11340722; doi:10.1136/bmjopen-2023-079338)
Supplement: Supplementary file 3 [file bmjopen-2023-079338.reviewer_comments.pdf]

PEER REVIEW HISTORY

BMJ Open publishes all reviews undertaken for accepted manuscripts. Reviewers are asked to complete a checklist review form (**http://bmjopen.bmj.com/site/about/resources/checklist.pdf**) and are provided with free text boxes to elaborate on their assessment. These free text comments are reproduced below.

ARTICLE DETAILS

**TITLE (PROVISIONAL)**	A systematic review of multi-variate time series approaches to extract predictive asthma biomarkers from prospectively patient-collected diary data
**AUTHORS**	Clemeno, Franz Aaron; Quek, Eleanor; Richardson, Matthew; Siddiqui, Salman

VERSION 1 – REVIEW

**REVIEWER**	Couillard, Simon<br>University of Oxford, Oxford Respiratory NIHR BRC, Nuffield Department of Medicine
**REVIEW RETURNED**	13-Sep-2023

**GENERAL COMMENTS**	Dear Authors,<br><br>Thank you for the opportunity to review your study which is essentially systematic review longitudinal monitoring through diary data (PEF ± FeNO) in asthma studies. A meta-analysis was impossible due to the heterogeneity of the studies and, indeed, you included multiple types of studies. Under that important caveat, your results section is mostly a description of the included studies, with no quantitative metric for pooling. Hence, I am very sorry to say that, in my sense, there is no novel result of interest besides a description of how diary cards were monitored and analysed. Nonetheless, here are areas for improvement, which I unfortunately I feel are impossible without formally repeating the work (#1) / doing a separate study (#2).<br><br>Comments<br>1. Your methodology relied on a mixed bag of RCTs, prospective/retrospective cohorts. Due to the intrinsic failures of real-world/observational cohorts, a case could be made to eliminate prospective/retrospective cohorts and only keep RCTs. Could this improve heterogeneity and thus allow a meta-analysis?<br>2. I thought the finding associating variability in control scores, PEF, and FeNO to exacerbation risk very interesting. Could you somehow derive a quantitative metric assessing this (e.g. entropy)? In the setting of the above change in systematic review inclusion criteria and more robust preliminary data along the lines of the first paragraphs of your results, a case could then be made use different RCTs placebo arms diary card results and analysing how these items longitudinally associate with risk.<br>3. The introduction would benefit from an introduction on the diary components / biomarkers of interest: i.e. defining PEF and FeNO and analysing their potential utilisation on a longitudinal basis.

**REVIEWER**	Chan, Amy<br>Auckland University
**REVIEW RETURNED**	10-Mar-2024

**GENERAL COMMENTS**	Well done on performing this systematic review of an interesting and very topical subject. Overall, the review is interesting and the findings will have important clinical and research relevance to further this emerging field to identify predictive asthma biomarkers from novel data sources and using innovative methodology. However, there are a few areas of concern that need addressing to ensure this is a clearly articulated and robust review for others to be able to replicate and refer to.<br><br>I have the following remarks which I hope will strengthen and clarify the review for others:<br>1) I am somewhat confused by the aim. The abstract refers to the aim being to evaluate methods for extracting biomarkers ...and investigate associations between the extracted measures and asthma PROs. However, when I read the results, it appears most of the Results and reported subheadings focus on the methods (e.g. threshold, ML/AI) of extracting biomarkers but then there is also a separate dedicated subheadings titled "biomarker extraction methods". Is threshold/ML/AI methods not a subcategory of biomarker extraction methods?<br>2) Abstract - could the authors add the date range of the search in the abstract?<br>3) I am a bit concerned about the overall comprehensiveness of the general search strategy - could the authors justify why only these 4 databases were searched and whether grey literature was included? It would seem to me that there may be more diary based /time series data that may be found in non-medical focused databases e.g. Web of Science or Scopus (see https://link.springer.com/article/10.1007/s11192-021-03948-5). I also wonder why the search was limited from 2000 onwards - what was the rationale?<br>4a) Inclusion /exclusion criteria: The review states that they focus on "studies that used routinely collected data in their analyses". I find this could be misleading as routinely collected data usually refers to electronic health records such as pharmacy claims data or GP records, which do not appear to be the focus of the review. I assume the authors mean they are referring to only prospectively collected diary data collected by the patient - is that correct? If so - I suggest this be clarified throughout the paper so readers know exactly how the authors decided what studies to include and what consitutes routinely collected diary variables (e.g. is a medication diary or electronic dispensing record considered a diary also?)<br>4b) The inclusion of only studies with data collected at least once daily over at least 2 weeks seems reasonable but how was this determined? Can the authors cite or refer to any other literature/research that provides the rationale for the once daily 2-week window as being sufficient temporal resolution and sampling duration to derive predictors? This is particularly important as this was the most common reason for excluding studies (n=24)<br>4c) The authors also refer to excluding "studies where the variables were beyond the scope of the review" - what variables would that be? It would be useful to understanding the authors' thinking on what variables were considered in scope versus those not in scope.<br><br>5) Whilst the review is an important one, I am concerned about this limitation that the authors refer to with only one author involved in screening. Could the authors clarify whether this was just screening or was only one author also involved in data extraction and study quality assessment? I would think this needs to be clarified in the Discussion

	and also article summary as this could be a key source of bias. More importantly, I am concerned this goes against best practice guidelines for undertaking systematic reviews - see here https://bmcmedresmethodol.biomedcentral.com/articles/10.1186/s12874-019-0782-0. I understand it can be difficult to resource double screening or extraction but at least 10% could be double screened or extracted? That wouldn't be an unmanageable amount - given the 1553 records, this would only be 155 for 10% or 310 for 20%? It would at least give better quality control, and likewise I'd recommend the authors consider the same for double extraction and reviewing of risk of bias. If not - then I suggest the authors seriously consider whether to downgrade this to a rapid review and not call this a systematic review?<br><br>6) Data extraction - how was the data extraction form created? Was this piloted? Could the authors also share what software they used for screening and extraction ?<br><br>7) Discussion - the authors refer to few studies that use ML/AI to build predictive models. I suggest the authors clarify that they mean the use of ML/AI with diary variables rather than with any variables for prediction as the number of ML/AI prediction studies are on the increase.<br><br>In terms of limitation - I would also include the exclusion of conference abstracts, and limit to the 4 databases as a limitation to be considered in the abstract.<br><br>The authors refer to 'entropy' - in the Discussion, could they clarify what this method is and whether they referred to this because there were studies in the review that used this method or did no studies use this method to date with diary variables?<br><br>The authors refer to a recommmendation that the review 'warrants use of remote sensors and electronic diaries ...' - but the review doesn't focus on either of these. Could you elaborate more on how your review of diary variables relate to these digital measures?<br><br>Minor - typo in line 456 - 'design shorts and more powerful' - I assume shorter...?<br>Suggest reviewing Discussion formatting and reducing the number of paragraph breaks - some paragraphs only have one or two sentences in it, whereas each paragraph should refer to a new idea and comprise a fuller body of sentences.<br>Figure 1 in the supplement is very useful. I suggest moving this to the main text

VERSION 1 – AUTHOR RESPONSE

Reviewer 1: We would like to thank the reviewer for their feedback and positive review of our manuscript and have responded to the comments raised below.

C1. Your methodology relied on a mixed bag of RCTs, prospective/retrospective cohorts. Due to the intrinsic failures of real-world/observational cohorts, a case could be made to eliminate prospective/retrospective cohorts and only keep RCTs. Could this improve heterogeneity and thus allow a meta-analysis?

R1. Thank you for this comment and suggestion – this amendment to the search strategy would indeed reduce trial heterogeneity and allow a meta-analysis, however, for this review, we wanted to

evaluate the overall utility of prospective diary variable collection beyond interventional trials. The high heterogeneity in the studies included is unfortunately a consequence of this aim.

C2. I thought the finding associating variability in control scores, PEF, and FeNO to exacerbation risk very interesting. Could you somehow derive a quantitative metric assessing this (e.g. entropy)? In the setting of the above change in systematic review inclusion criteria and more robust preliminary data along the lines of the first paragraphs of your results, a case could then be made use different RCTs placebo arms diary card results and analysing how these items longitudinally associate with risk.

R2. We acknowledge that single quantitative metric that summarises the associations between the diary variables and the asthma PROs would be ideal and would be very helpful to assess the relationships between the two. However, this would be more fitting for a further study that focuses particularly on diary variables within the context solely of RCTs and interventional trials.

C3. The introduction would benefit from an introduction on the diary components / biomarkers of interest: i.e. defining PEF and FeNO and analysing their potential utilisation on a longitudinal basis.

R3. A brief overview of each of the diary variables of interest has been added to the Introduction section (see lines 86-107).

Reviewer 2: We would like to thank the reviewer for their feedback and positive review of our manuscript and have responded to the comments raised below.

C1. The abstract refers to the aim being to evaluate methods for extracting biomarkers ...and investigate associations between the extracted measures and asthma PROs. However, when I read the results, it appears most of the Results and reported subheadings focus on the methods (e.g. threshold, ML/AI) of extracting biomarkers but then there is also a separate dedicated subheadings titled "biomarker extraction methods". Is threshold/ML/AI methods not a subcategory of biomarker extraction methods?

R1. The subheadings in the Results section have been changed. Specifically, the threshold, ML and AI methods have been included under “Biomarker extraction methods”.

C2. Abstract - could the authors add the date range of the search in the abstract?

R2. The date range of the search has been added to the abstract (see lines 40-41).

C3. I am a bit concerned about the overall comprehensiveness of the general search strategy - could the authors justify why only these 4 databases were searched and whether grey literature was included? I also wonder why the search was limited from 2000 onwards - what was the rationale?

R3. The choice for the search range was influenced by the increased interest and implementation of digital health and digital remote patient monitoring, which was not until the early 2000s (DOI: 10.1109/IEMBS.2003.1279581). The choice of the databases was due to the primary medical focus of the time series analysis; we wanted to evaluate how time series in asthma specifically were analysed and how its behaviour related to various outcomes. We acknowledge that some studies may have been missed and have included searching only the four databases as a limitation to the systematic review (see lines 500-501).

C4. Inclusion /exclusion criteria: The review states that they focus on "studies that used routinely collected data in their analyses". I find this could be misleading as routinely collected data usually refers to electronic health records such as pharmacy claims data or GP records, which do not appear to be the focus of the review. I assume the authors mean they are referring to only prospectively collected diary data collected by the patient - is that correct? If so - I suggest this be clarified throughout the paper so readers know exactly how the authors decided what studies to include and what constitutes routinely collected diary variables (e.g. is a medication diary or electronic dispensing record considered a diary also?)

R4. The terminology has been changed in the manuscript, from "routinely collected diary variables" to "prospective patient-collected diary variables". Diary variables in this study refer to PEF, symptom scores, reliever use, and nocturnal awakenings, although we acknowledge that other variables may be collected in a patient diary also.

C5. The inclusion of only studies with data collected at least once daily over at least 2 weeks seems reasonable but how was this determined? Can the authors cite or refer to any other literature/research that provides the rationale for the once daily 2-week window as being sufficient temporal resolution and sampling duration to derive predictors? This is particularly important as this was the most common reason for excluding studies (n=24)

R5. The justification for the follow-up duration has been added to the manuscript (see lines 165-171). A previous study suggests that in fractal analyses of time series, there is an increased risk of bias in time series that are shorter than 64 data points. A more relaxed criterion was used for this review as to not to limit the included studies. However, only one of the included studies had a follow-up duration of two weeks (roughly 30 data points), with the rest of the studies having more than 64 data points in their data. The data collection frequency used in the systematic review also mirrors the guideline recommendations for short-term PEF monitoring for determining baseline values in asthma action plans.

C6. The authors also refer to excluding "studies where the variables were beyond the scope of the review" - what variables would that be? It would be useful to understanding the authors' thinking on what variables were considered in scope versus those not in scope.

R6. Some clarification regarding what variables were regarded as “beyond the scope” has been added (see lines 211-213). These included studies that analysed airway impedance from impulse oscillometry, FEV1, IL-4, IL-5, and IL-13. In other words, these studies did not investigate associations between diary variables (namely PEF, symptoms, reliever use, awakenings) and asthma PROs (asthma exacerbations, asthma control and asthma-related quality of life) of interest.

C7. Whilst the review is an important one, I am concerned about this limitation that the authors refer to with only one author involved in screening. Could the authors clarify whether this was just screening or was only one author also involved in data extraction and study quality assessment? I would think this needs to be clarified in the Discussion and also article summary as this could be a key source of bias. I understand it can be difficult to resource double screening or extraction but at least 10% could be double screened or extracted? It would at least give better quality control, and likewise I'd recommend the authors consider the same for double extraction and reviewing of risk of bias.

R7. We acknowledge that having a single reviewer introduces significant potential bias in the search process. Thus, a secondary reviewer has now been involved and has screened 10% of the titles and abstracts. Additionally, the secondary reviewer performed the data extraction and quality assessment of 10% of the included full-text articles, reducing the potential bias in the overall search methodology and quality assessment. This has been added to the manuscript.

The secondary reviewer has been added as a co-author to the manuscript (E. Quek).

Addition of a secondary reviewer has been added in lines 178-179.

Significant changes from the data extraction of the secondary reviewer were changed in Supplementary Table 1.

There were no differences in the outcomes of the TRIPOD and PROBAST assessments and so no changes needed to be made.

C8. Data extraction - how was the data extraction form created? Was this piloted? Could the authors also share what software they used for screening and extraction?

R8. Further clarification has been added into the manuscript (see lines 186-191). The data extraction form was initially piloted, and the extraction process was conducted manually. The title and abstract screening were performed using Rayyan.

C9. Discussion - the authors refer to few studies that use ML/AI to build predictive models. I suggest the authors clarify that they mean the use of ML/AI with diary variables rather than with any variables for prediction as the number of ML/AI prediction studies are on the increase.

R9. This point has been clarified in the manuscript; we refer to there being few studies using ML/AI with diary variables for event prediction in asthma, rather than the general use of ML/AI (see lines 413-414).

C10. In terms of limitation - I would also include the exclusion of conference abstracts, and limit to the 4 databases as a limitation to be considered in the abstract.

R10. We acknowledge these as limitations have to the study and these have been added to the manuscript (see lines 500-501).

C11. The authors refer to 'entropy' - in the Discussion, could they clarify what this method is and whether they referred to this because there were studies in the review that used this method or did no studies use this method to date with diary variables?

R11. A short description of entropy has been added in the Discussion section (see lines 451-459). We also clarified that no published studies to date have evaluated the associations between the entropy of diary variables with asthma PROs. Entropy however has been applied to other variables in asthma (e.g., respiratory impedance and airway resistance) and are potential predictors of asthma progression and asthma severity.

C12. The authors refer to a recommendation that the review 'warrants use of remote sensors and electronic diaries ...' - but the review doesn't focus on either of these. Could you elaborate more on how your review of diary variables relate to these digital measures?

R12. A short paragraph regarding mHealth and its potential use in asthma has been added to the introduction. The increase in digital patient measurement gives rise to increased availability of diary variables for clinicians and researchers, opening the possibility and need for statistical models that can leverage these data into predictive models and more efficient self-management plans. This study showed that predictive biomarkers can be derived from prospectively collected diary variables, thus showing a potential use for mHealth for early event prediction in asthma.

An introduction to mHealth was added in lines 128-136

The implication of the results to mHealth uses was added in lines 502-507

C13. Minor - typo in line 456 - 'design shorts and more powerful'

R13. This typo has been fixed (see line 510).

C14. Suggest reviewing Discussion formatting and reducing the number of paragraph breaks - some paragraphs only have one or two sentences in it, whereas each paragraph should refer to a new idea and comprise a fuller body of sentences.

R14. The number of paragraph breaks have been reduced in the Discussion section as per the comment.

C15. Figure 1 in the supplement is very useful. I suggest moving this to the main text

R15. Supplementary figure 1 has been moved into the main text and is now labelled as Figure 5.

VERSION 2 – REVIEW

**REVIEWER**	Couillard, Simon<br>University of Oxford, Oxford Respiratory NIHR BRC, Nuffield Department of Medicine
**REVIEW RETURNED**	08-May-2024

**GENERAL COMMENTS**	No further comment. Congratulations.

**REVIEWER**	Chan, Amy<br>Auckland University
**REVIEW RETURNED**	05-Jun-2024

**GENERAL COMMENTS**	Thank you for the opportunity to review this again. The authors have significantly improved the manuscript, taken time to address the comments and ensuring that at least 10% of the abstracts and data have been double screened and checked. The language has been updated to more clearly capture the aims of this review. Well done.
